# Communities for Healthy Living (CHL) A Community-based Intervention to Prevent Obesity in Low-Income Preschool Children: Process Evaluation Protocol

**DOI:** 10.1186/s13063-020-04571-0

**Published:** 2020-07-23

**Authors:** Jacob P. Beckerman-Hsu, Alyssa Aftosmes-Tobio, Adam Gavarkovs, Nicole Kitos, Roger Figueroa, Z. Begum Kalyoncu, Kindra Lansburg, Xinting Yu, Crystal Kazik, Adrienne Vigilante, Jessie Leonard, Merieka Torrico, Janine M. Jurkowski, Kirsten K. Davison

**Affiliations:** 1 Department of Nutrition, Harvard T.H. Chan School of Public Health, 677 Huntington Ave, Boston, MA 02115 USA; 2 Boston College School of Social Work, McGuinn Hall Room 115, 140 Commonwealth Ave, Chestnut Hill, MA 02467 USA; 3 Institute of Health Policy, Management and Evaluation & Wilson Centre, Faculty of Medicine, University of Toronto, Toronto, Canada; 4 Massachusetts Department of Public Health, Boston, MA 02115 USA; 5 Division of Nutritional Sciences, Cornell University, 244 Garden Ave, Ithaca, NY 14853 USA; 6 Nutrition and Dietetics Department, Atilim University, Kizilcasar Mahallesi, Incek Golbasi, 06830 Ankara, Turkey; 7 Action for Boston Community Development, 178 Tremont Street, Boston, MA 02111 USA; 8 Departments of Medicine, Brigham and Women’s Hospital and Beth Israel Deaconess Medical Center, Harvard Medical School, Boston, MA 02115 USA; 9 Community Action Agency of Somerville, 66 Union Square, Somerville, MA 02143 USA; 10 Department of Health Policy, Management & Behavior, University at Albany School of Public Health, 1 University Place, Rensselaer, NY 12144 USA

**Keywords:** Process evaluation, Community-based participatory research, Adaptive intervention, Stepped-wedge trial

## Abstract

**Background:**

Process evaluation can illuminate barriers and facilitators to intervention implementation as well as the drivers of intervention outcomes. However, few obesity intervention studies have documented process evaluation methods and results. Community-based participatory research (CBPR) requires that process evaluation methods be developed to (a) prioritize community members’ power to adapt the program to local needs over strict adherence to intervention protocols, (b) share process evaluation data with implementers to maximize benefit to participants, and (c) ensure partner organizations are not overburdened. Co-designed with low-income parents using CBPR, Communities for Healthy Living (CHL) is a family-centered intervention implemented within Head Start to prevent childhood obesity and promote family well-being. We are currently undertaking a randomized controlled trial to test the effectiveness of CHL in 23 Head Start centers in the greater Boston area. In this protocol paper, we outline an embedded process evaluation designed to monitor intervention adherence and adaptation, support ongoing quality improvement, and examine contextual factors that may moderate intervention implementation and/or effectiveness.

**Methods:**

This mixed methods process evaluation was developed using the Pérez et al. framework for evaluating adaptive interventions and is reported following guidelines outlined by Grant et al. Trained research assistants will conduct structured observations of intervention sessions. Intervention facilitators and recipients, along with Head Start staff, will complete surveys and semi-structured interviews. De-identified data for all eligible children and families will be extracted from Head Start administrative records. Qualitative data will be analyzed thematically. Quantitative and qualitative data will be integrated using triangulation methods to assess intervention adherence, monitor adaptations, and identify moderators of intervention implementation and effectiveness.

**Discussion:**

A diverse set of quantitative and qualitative data sources are employed to fully characterize CHL implementation. Simultaneously, CHL’s process evaluation will provide a case study on strategies to address the challenges of process evaluation for CBPR interventions. Results from this process evaluation will help to explain variation in intervention implementation and outcomes across Head Start programs, support CHL sustainability and future scale-up, and provide guidance for future complex interventions developed using CBPR.

**Trial registration:**

ClinicalTrials.gov, NCT03334669. Registered on October 10, 2017

## Background

Community-based obesity interventions require careful process evaluation, as their many component parts can be implemented with varying degrees of completeness and quality. Yet, few obesity interventions publish comprehensive process evaluation methods and results [1, 2]. Without capturing variation in implementation, it is impossible to accurately attribute results to any given aspect of the intervention or identify intervention elements appropriate for scale-up [3]. Process evaluation is also critical for understanding facilitators and barriers to successful intervention implementation. Evaluating the intervention’s “viable validity” [4], or the feasibility of successfully implementing and sustaining the intervention, is critical for intervention scale-up and sustainability.

Community-based participatory research (CBPR) interventions are a special case of community interventions in which the intended program recipients are equal partners with researchers and other community members in intervention development and implementation [5]. CBPR interventions present three main process evaluation challenges compared to researcher-oriented interventions. First, the CBPR approach calls into question the traditional focus on fidelity to intervention protocols [6], also known as adherence. Focusing on adherence prioritizes the decisions of intervention designers over implementers’ power to adapt the intervention to meet local needs [7], thereby violating the CBPR principle of mutual empowerment of all involved [5]. Measuring both adherence and adaptations better aligns to the ethos of CBPR. Moreover, measuring adaptation is vital given that interventions are more often adapted than not [7, 8].

The second challenge of process evaluation in CBPR is that process evaluation efforts are traditionally designed to be entirely separate from the intervention itself. While this separation can strengthen understanding of intervention effects in the absence of process evaluation, it comes at the cost of bypassing opportunities to use process evaluation data to improve program implementation, which could benefit participants. This trade-off is not appropriate in CBPR projects, which require mutual benefits for all partners [5]. Using process evaluation data during the intervention can also increase the likelihood of producing sustainable and effective interventions [9].

The final process evaluation challenge in CBPR also concerns mutual benefit for all partners. When community partners are responsible for implementing interventions, as is frequently the case in CBPR, data collection must not impede partners’ ability to deliver high-quality services to the community. Hence, utilizing existing data sources for process evaluation is critical, and it may be necessary to forego some aspects of process evaluation that would overburden community partners.

To address the dearth of process evaluation reporting in the obesity intervention literature in general and CBPR interventions in particular, this paper outlines the pre-specified process evaluation for Communities for Healthy Living (CHL) integrated into the main trial. CHL is a complex intervention, originally developed in collaboration with low-income parents using CBPR, to prevent obesity in preschool-aged children enrolled in Head Start. Following process evaluation guidelines outlined by Grant et al. [10] and expanding on a brief description of CHL’s process evaluation in the main trial protocol [11], this protocol paper provides comprehensive information on CHL’s implementation, adaptation, and context. Results from the process evaluation will aid the interpretation of the main trial results and inform future scale-up efforts. Specific objectives are as follows:

1. Assess intervention adherence (including intervention content, reach and duration, quality of delivery, participant representativeness and responsiveness) and variation in adherence across Head Start programs
2. Inform and document CHL adaptations and quality improvement strategies
3. Examine setting-specific factors (such as perceived readiness to implement, anticipated benefits, perceived importance and relevance of the intervention, coaching support received, and organizational capacity) that may moderate intervention adherence or effectiveness
4. Outline strategies to address the challenges of process evaluation for CBPR interventions, which are seldom reported in CBPR or other obesity prevention efforts [12–19], including strategies for (a) measuring adherence and adaptation, (b) utilizing process evaluation data for quality improvement, and (c) balancing the benefits and burdens of data collection for intervention implementers.

## Methods

### Process evaluation reporting protocol

Our reporting of CHL’s process evaluation protocol is informed by the reporting framework for process evaluations for cluster-randomized trials of complex interventions outlined by Grant and colleagues [10]. While there is tremendous variation in the design and structure of process evaluations, thus necessitating flexibility in their reporting, Grant et al. [10] specify that all process evaluation protocols should (a) be clearly labeled, (b) state their purpose, (c) report if they were pre-specified or post hoc, (d) state the choice of methods and justify them, and (e) summarize or refer to the main trial protocol.

### Communities for Healthy Living

CHL is an early childhood obesity prevention program that is currently being evaluated in a cluster-randomized type I hybrid [20] stepped-wedge trial. CHL will be implemented in 16 Head Start programs, which encompass 23 individual centers, throughout Boston, Cambridge, and Somerville, MA, from fall 2017 to spring 2020. A comprehensive description of the intervention and trial design is presented in the main protocol paper [11]. As summarized briefly below, the intervention has three main components including (a) the Parents Connect for Healthy Living (PConnect) program, (b) enhanced nutrition support, and (c) a media campaign.

The *PConnect* program is a 10-week, in-person health and empowerment class open to all parents and primary caregivers whose children attend Head Start programs assigned to the intervention. Each PConnect program is led by a Head Start staff member and parent who work together as co-facilitators. Prior to implementing PConnect, all facilitators complete a 3-day training on program content and facilitation skills. During PConnect, facilitators meet weekly with a quality improvement coach (typically a research team member involved in creating the PConnect program), who helps them reflect on the past session and prepare for the next session.

*Enhanced nutrition support* includes resources and protocols designed to increase Head Start’s effectiveness in communicating with families about child health. Per Head Start performance standard 1302.33 [21], study area Head Start programs already perform biannual health screenings and shares results with families in a Health and Growth Letter. As a part of the CHL intervention, two new letters were created: a “primer letter” and a revised Health and Growth Letter. Before sending results of the standard Head Start health screenings, programs in the intervention first distribute the primer letter, which explains to families what is included in the upcoming Health and Growth Letter (e.g., their child’s height and weight measurement) and staff contact information if families have questions or concerns. Then, intervention programs send parents the revised Health and Growth Letter, which was redesigned to clearly communicate health screening results and highlight steps that parents can take to help their children have healthy behaviors. Enhanced nutrition support also includes training sessions and resources to bolster the ability of Head Start staff to discuss child health and nutrition with parents.

The *media campaign* includes (a) printed educational materials about nutrition, consumption of healthy drinks (and avoidance of sugary drinks), physical activity, sleep, and limiting screen time, all of which promote healthy child weight and are collectively referred to as the “Healthy Habits”; (b) posters and flyers to promote CHL and the Healthy Habits in Head Start centers; and (c) a web-based Neighborhood Resource Map, which helps families find local resources to support them with the Healthy Habits.

### An evaluation framework to capture adherence and adaptation

Adaptive interventions are designed to permit or even encourage changes during implementation based on context-specific implementer and participant needs [7], and as such, align with CBPR principles [5]. Pérez et al. [7] outline three important domains to assess during the process evaluation of an adaptive intervention: adherence, adaptation, and moderators. For each of the CHL intervention components, we designed measures to capture all domains and subdomains of this process evaluation framework. A summary of the data collection tools used and the indicators for each domain can be found in Tables 1 and 2, respectively. Definitions of each process evaluation domain and subdomain follow with illustrative examples using the PConnect program.

**Table 1:** Data sources employed in CHL process evaluation

Data source	Collected from	Collection frequency (time)	Process evaluation data collected	Administration mode	CHL^**1**^ component(s) evaluated
**Surveys**					
A. PConnect^2^ training evaluation	PConnect^2^ facilitators	Annual (end of PConnect^2^ training, typically March)	• Training effectiveness • Training quality • PConnect^2^ program and materials quality	Paper	• PConnect^2^
B. PConnect^2^ evaluation	PConnect^2^ participants	Annual (at end of PConnect^2^)	• Quality of PConnect^2^ content • Quality of PConnect^2^ materials • Quality of PConnect^2^ facilitation	Paper	• PConnect^2^
C. Staff training evaluation	Head Start staff (teachers, family advocates)	Annual (fall)	• Clarity/complexity of staff role in CHL • Self-efficacy for using CHL resources	Paper	• Enhanced nutrition support • Media campaign
D. Head Start staff survey	Head Start staff (health/nutrition, teachers, family advocates, administrators)	Biannual (fall, spring)	• Staff readiness to implement intervention • Staff use of CHL materials • Staff-perceived quality and usefulness of CHL materials	Paper, electronic	• Enhanced nutrition support • Media campaign
E. Parent outcomes survey	Head Start parents (convenience sample)	Annual (spring)	• Parent experiences with CHL	Paper, electronic	• PConnect^2^ • Enhanced nutrition support • Media campaign
**Interviews and focus groups**					
F. PConnect^2^ facilitator interviews	PConnect^2^ facilitators	Annual (after PConnect^2^ ends)	• Adequacy of training, quality improvement coaching, and materials • Demands on facilitators • Impacts on facilitators (e.g., health behaviors, empowerment) • Appropriateness and acceptability of parent-staff co-facilitation model • Number of participants using social media and content of posts made	In-person, phone	• PConnect^2^ • Enhanced nutrition support
G. PConnect^2^ participant interviews	PConnect^2^ participants (two randomly selected per PConnect^2^ program)	Annual (after PConnect^2^ ends)	• Impacts on participants (e.g., health behaviors, empowerment) • Appropriateness and acceptability of parent-staff co-facilitation model • Facilitation quality • Number of participants using social media and content of posts made	In-person, phone	• PConnect^2^
H. Head Start staff interviews and focus groups	Head Start staff (program directors, family engagement, health/nutrition)	Annual (spring)	• Barriers and facilitators to CHL implementation • Effects of CHL on Head Start as a whole	Phone (interviews), in-person (focus groups)	• PConnect^2^ • Enhanced nutrition support • Media campaign
**Administrative records**					
I. Administrative records	Varied	Varied	• Attendance • Material distribution	Paper, electronic	• PConnect^2^ • Enhanced nutrition support • Media campaign
**Website analytics**					
J. Website analytics	Neighborhood Resource Map website (accessible only to intervention group staff and parents)	Continuous	For each website visit: • Device-specific ID • Date • Time of day	Electronic	• Media campaign
K. Neighborhood Resource Map database	Neighborhood Resource Map website (accessible only to intervention group staff and parents)	Continuous	Changes requested to Neighborhood Resource Map	Electronic	• Media campaign
**PConnect observations and checklists**					
L. PConnect^2^ session checklists	PConnect^2^ facilitators	One per PConnect^2^ session/program/year	For each session: • Activities completed • Activities modified	Paper	• PConnect^2^
M. PConnect^2^ coaching reflection sheet	PConnect^2^ facilitators and PConnect^2^ quality improvement coach	One per PConnect^2^ session/program/year	• Adherence • Adaptations • Facilitation quality • Participant responsiveness	Paper	• PConnect^2^
N. PConnect^2^ session observations	Trained observer	Annual (one session observed per PConnect^2^ program)	• Adherence • Adaptations • Facilitation quality • Participant responsiveness	Paper	• PConnect^2^

Mixed methods will be applied to create a full understanding of CHL implementation

^1^Communities for Healthy Living

^2^Parents Connect for Healthy Living

**Table 2:** Process evaluation indicators and data sources for each Communities for Healthy Living (CHL) intervention component

Domain and subdomain			Communities for Healthy Living intervention components		
			Parents Connect for Healthy Living (PConnect)	Enhanced nutrition support	Media campaign
Adherence	Content		- Activities completed during each session (L)	- Content of primer letter, revised Health and Growth Letter (I) - Content of training (I)	- Content of brochures, posters, and Neighborhood Resource Map (I, K)
	Reach		- No. of parents attending (I)	- No. of families sent primer letter + revised Health and Growth Letter (I) - No. of staff trained (I)	- No. of CHL print materials distributed (I) - No. of Neighborhood Resource Map visits (J)
	Frequency		- Session date (I) - Session duration (L)	- Date of distribution of primer letter, revised Health and Growth Letter (I) - Date and duration of Head Start staff training (I)	- Date of Neighborhood Resource Map visits (J)
	Duration				
Adaptation	Additions, deletions, modifications		- Changes to session content or duration (L, M, N)	- Changes to training frequency and duration(I)	- Changes in brochures, posters, or Neighborhood Resource Map (I, K)
Moderators	Quality of intervention delivery		- Participant- (B, G), facilitator- (F, M), and trained observer (N)-perceived quality of PConnect facilitation	- Quality of training (C)	n/a
	Participant responsiveness		- Participant-(B, G), facilitator-(M, F), and trained observer (N)-reported participant responsiveness - Number, frequency, and content of parent interactions on PConnect social media (G, F)	- Parent recall of receipt and contents of revised Health and Growth Letter (E) - Staff-perceived helpfulness of CHL resources (D) - No. of staff who use skills and/or resources from training (D)	- Parent recall of receipt of brochures, flyers (E) - Parent-reported usefulness of brochures (B) - Staff-reported parent engagement with posters/flyers (D, H)
	Barriers and facilitators to implementation	Intervention description	- Facilitator-perceived clarity and complexity of PConnect (A, F, M) - Adequacy of PConnect facilitator training (A, F) - Adequacy of support provided during PConnect (F)	- Staff-perceived clarity and complexity of their role in CHL (C, H)	n/a
		Situation- and setting-specific factors	- Situation- and setting-specific factors influencing PConnect implementation (F, G, H, M, N)	- Situation- and setting-specific factors influencing training and/or resource use (H)	- Situation- and setting-specific factors influencing brochure distribution, putting up posters, and/or demonstrations of the Neighborhood Resource Map (H)

Letters in parenthesis refer to data sources listed in Table 1. A, Parents Connect for Healthy Living (PConnect) training evaluation; B, PConnect evaluation; C, staff training evaluation; D, Head Start staff survey; E, parent outcomes survey; F, PConnect facilitator interviews; G, PConnect participant interviews; H, Head Start staff interviews and focus groups; I, administrative records; J, website analytics; K, Neighborhood Resource Map database; L, PConnect session checklists; M, PConnect coaching reflection sheet; N, PConnect session observations

*Adherence* describes the degree to which the intervention is implemented per original protocols. Important subdomains of adherence include the content of the intervention (e.g., were the PConnect session outlines followed?), reach (e.g., how many people attended PConnect? are participants representative of the target population?), frequency (e.g., was PConnect offered as often as planned?), and duration (e.g., did each PConnect session last for the planned amount of time?).

*Adaptation* describes any deviation from an intervention as originally planned [7]. Consistent with the Rebchook et al. [22] system for categorization, adaptations will be characterized as additions (e.g., was an extra handout or resource shared during a PConnect session?), deletions (e.g., was a portion of a PConnect session skipped?), and modifications (e.g., was an activity completed individually instead of as a group?).

*Intervention moderators* are factors that can change intervention implementation and/or effectiveness and include (a) the quality of intervention delivery (e.g., the use of appropriate pedagogical strategies), (b) the participant responsiveness (e.g., amount of participation in PConnect social media), (c) the comprehensiveness of intervention description (e.g., the clarity and complexity of PConnect session outlines), and (d) the setting- or situation-specific moderators (e.g., not being able to hang any posters because PConnect is held in a shared space). It is important to note a key difference between these four subdomains described by Pérez et al. [7]. The first two factors, quality of intervention delivery and participant responsiveness, describe implementation of the intervention as it occurred; these data can help to explain intervention outcomes. For example, perhaps Head Start programs where CHL is implemented with higher quality (e.g., better PConnect facilitation) will have greater improvement in intervention outcomes. The second two factors, on the other hand, provide insight as to *why* the intervention may have been implemented as it was. For instance, PConnect leaders would have a difficult time delivering the sessions well if the PConnect training did not adequately explain the content of the sessions (intervention description). As one of the aims of this paper is to highlight the utility of process evaluation for characterizing the viable validity [4] of interventions, intervention description and setting- or situation-specific moderators will be discussed collectively as barriers and facilitators to implementation. Understanding these barriers and facilitators to implementation can be used to identify ways to sustain the intervention in programs seeking to continue CHL, and for new programs added during intervention scale-up.

### Process evaluation for each CHL component

As summarized in Table 1, CHL process evaluation data are compiled across a wide range of qualitative and quantitative data sources including (1) surveys completed by Head Start parents and staff, including those facilitating and participating in PConnect; (2) semi-structured interviews conducted with PConnect participants, PConnect facilitators, and Head Start staff; (3) focus groups with Head Start staff; (4) administrative records; (5) social media and website analytics; and (6) observations of PConnect sessions. The integration of qualitative and quantitative methods ensures that a broad cross-section of intervention facilitators and implementers is represented and that concrete implementation factors such as attendance are measured, while simultaneously capturing in-depth information on respondent experiences and setting-specific factors. As summarized in Table 2 and in the “Adherence” to “Quality improvement” sections below, data from these sources will be used to characterize adherence, adaptation, and moderators of implementation and effectiveness for all components of CHL. For PConnect, process evaluation data will also be used for quality improvement.

The process evaluation for the PConnect program is at the parent level and focuses on parent participants and the facilitators who lead the program. Process evaluation for enhanced nutrition support and the media campaign, on the other hand, focuses on program-level factors. This decision has both theoretical and pragmatic motivations. Both enhanced nutrition support and the media campaign are conceptualized as Head Start program-level interventions meant to achieve organizational empowerment by enhancing the capacity of the programs to promote health among the families they serve [11]. Therefore, the process evaluation for these components includes how staff are trained and the resources given to programs. From a pragmatic standpoint, since staff are responsible for implementing enhanced nutrition support and the media campaign, detailed data collection on how these intervention elements are delivered to parents would place substantial burden on staff. As such, there is only limited process evaluation at the parent level for enhanced nutrition support and the media campaign.

#### Adherence

Adherence to the intervention protocol for the PConnect program is operationalized as the number of planned PConnect activities implemented at each session (data sources: session checklists, coaching reflection sheets, observations), the participant attendance (data source: administrative records), the number of PConnect sessions implemented each year (data source: administrative records), and the duration of PConnect sessions (data source: session checklists) (Tables 1 and 2).

For enhanced nutrition support, adherence incorporates the number of families who are sent the primer letter and the revised Health and Growth Letter in the fall and spring (data source: administrative records), the content of staff training on enhanced nutrition support procedures and resources (data source: administrative records), the number of relevant staff trained in the enhanced nutrition support procedures (data source: administrative records), and the frequency and duration of the staff training sessions (data source: administrative records) (Tables 1 and 2).

Adherence to the media campaign includes the number of CHL print materials distributed (data source: administrative records), the number of online Neighborhood Resource Map visits (data source: website analytics), and the date of online Neighborhood Resource Map visits (data source: website analytics) (Tables 1 and 2).

#### Adaptation

Many adaptations to PConnect are expected; some will be conscious decisions that facilitators make to more effectively meet the needs of parents in specific communities, and others unintentional (e.g., running out of time for an activity). Additions, deletions, and modifications of sessions are captured on session checklists and coaching reflection sheets (Tables 1 and 2).

No adaptations are expected for the content of printed materials used in enhanced nutrition support and the media campaign, as the Community Advisory Board comprised of Head Start staff, teachers, and parents thoroughly reviewed these materials before the intervention began [11]. Modifications to the frequency and/or duration of enhanced nutrition support trainings are documented in administrative records. The content of the Neighborhood Resource Map, on the other hand, is expected to change as Head Start parents and staff request additions, deletions, and modifications. The Neighborhood Resource Map database stores all changes made (Tables 1 and 2).

#### Moderators: quality of intervention delivery, participant responsiveness

The quality of PConnect facilitation is assessed by participants (data sources: PConnect evaluation, parent interviews), the facilitators themselves (data sources: coaching reflection sheets, facilitator interview), and trained members of the CHL research team (data source: observation). Parent responsiveness to PConnect is reported by the parent participants (data sources: PConnect evaluation, parent interviews), facilitators (data sources: coaching reflection sheets, facilitator interview), and trained members of the CHL research team (data source: observations). A final measure of participant responsiveness collected is the number of parents using social media and the content of posts made (data sources: parent and facilitator interviews) (Tables 1 and 2).

For enhanced nutrition support, the quality of intervention delivery is operationalized as staff-perceived quality of enhanced nutrition support training (data source: staff training evaluation). Participant responsiveness includes staff-perceived usefulness of enhanced nutrition support resources (data source: staff survey), the number of staff who use skills and/or resources from their enhanced nutrition support training (data source: staff survey), and parent recall of the revised Health and Growth Letter (data source: parent outcomes survey).

For the CHL media campaign, parent responsiveness is assessed by parents and staff (data sources: parent outcomes survey, staff survey, staff interview). As this aspect of the intervention only requires distribution of materials, quality of intervention delivery was not a relevant subdomain to assess (Tables 1 and 2).

#### Moderators: barriers and facilitators to implementation

Barriers and facilitators to PConnect implementation are primarily reported by PConnect facilitators. Facilitator perception of the clarity and complexity of PConnect is assessed at three time points: upon completing the facilitator training (data source: PConnect training evaluation), during the PConnect program (data source: coaching reflection sheets), and after the PConnect program (data source: facilitator interview). Facilitators also evaluate the adequacy of the facilitator training (data sources: PConnect training evaluation, facilitator interview) and the coaching throughout the program (data source: facilitator interview). To capture barriers and facilitators outside the control of the PConnect facilitators, qualitative data on situation- and setting-specific factors that affect implementation are recorded by the PConnect facilitators, participants, observers, and Head Start staff (data sources: coaching reflection sheet, facilitator interviews, participant interviews, observations, Head Start staff interviews). These factors can range from the quality of the classroom in which PConnect is held to the degree of cultural adaptation required for PConnect to be successful in a given community (Tables 1 and 2).

Barriers and facilitators to implementing enhanced nutrition support are measured at the staff and program levels. Staff evaluate the clarity and complexity of enhanced nutrition support (data source: staff survey) and describe any assistance they receive or challenges they face in providing enhanced nutrition support (data sources: staff survey, staff interview) (Tables 1 and 2).

Staff also assess barriers and facilitators to implementing the media campaign, including the ability of Head Start programs to distribute print materials to parents, display CHL media in their centers, and help parents use web-based CHL media (data source: interview). Because distribution of these media campaign materials was so straightforward, it was not relevant to assess the intervention description subdomain for the media campaign (Tables 1 and 2).

#### Quality improvement

**Fig. 1:**
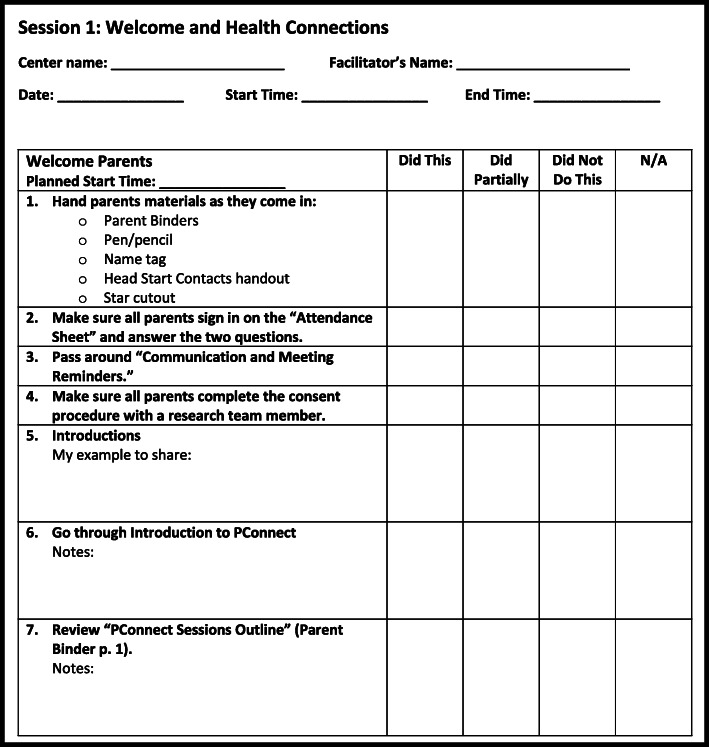
Sample page from a Parents Connect for Healthy Living (PConnect) session checklist. Each of the 10 PConnect sessions has a checklist to guide facilitators during the session. Facilitators prepare for sessions by making notes of how they will lead each activity and preparing examples to share. During and immediately after the session, facilitators check off what they did as originally planned and what they changed

**Fig. 2:**
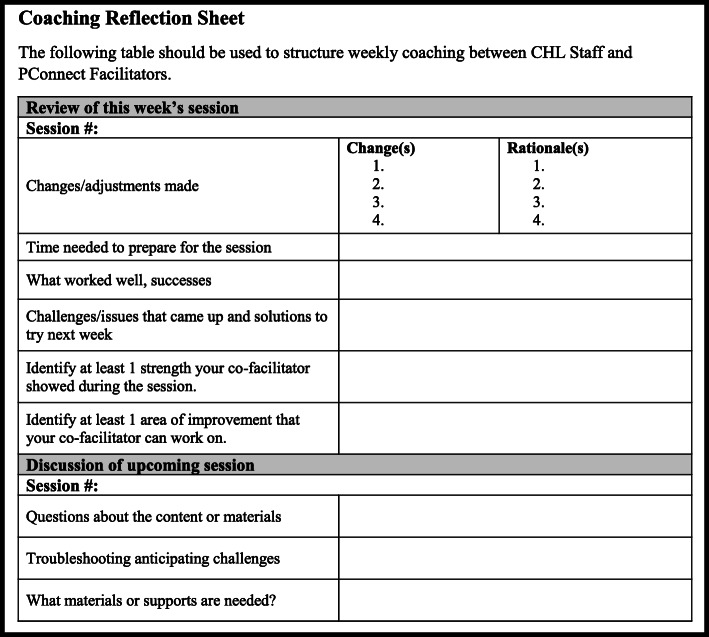
Parents Connect for Healthy Living (PConnect) coaching reflection sheet. This outline is used to structure the weekly coaching that helps PConnect facilitators reflect on each session and make strategic changes to their facilitation strategies to maximize program effectiveness. CHL, Communities for Healthy Living

**Fig. 3:**
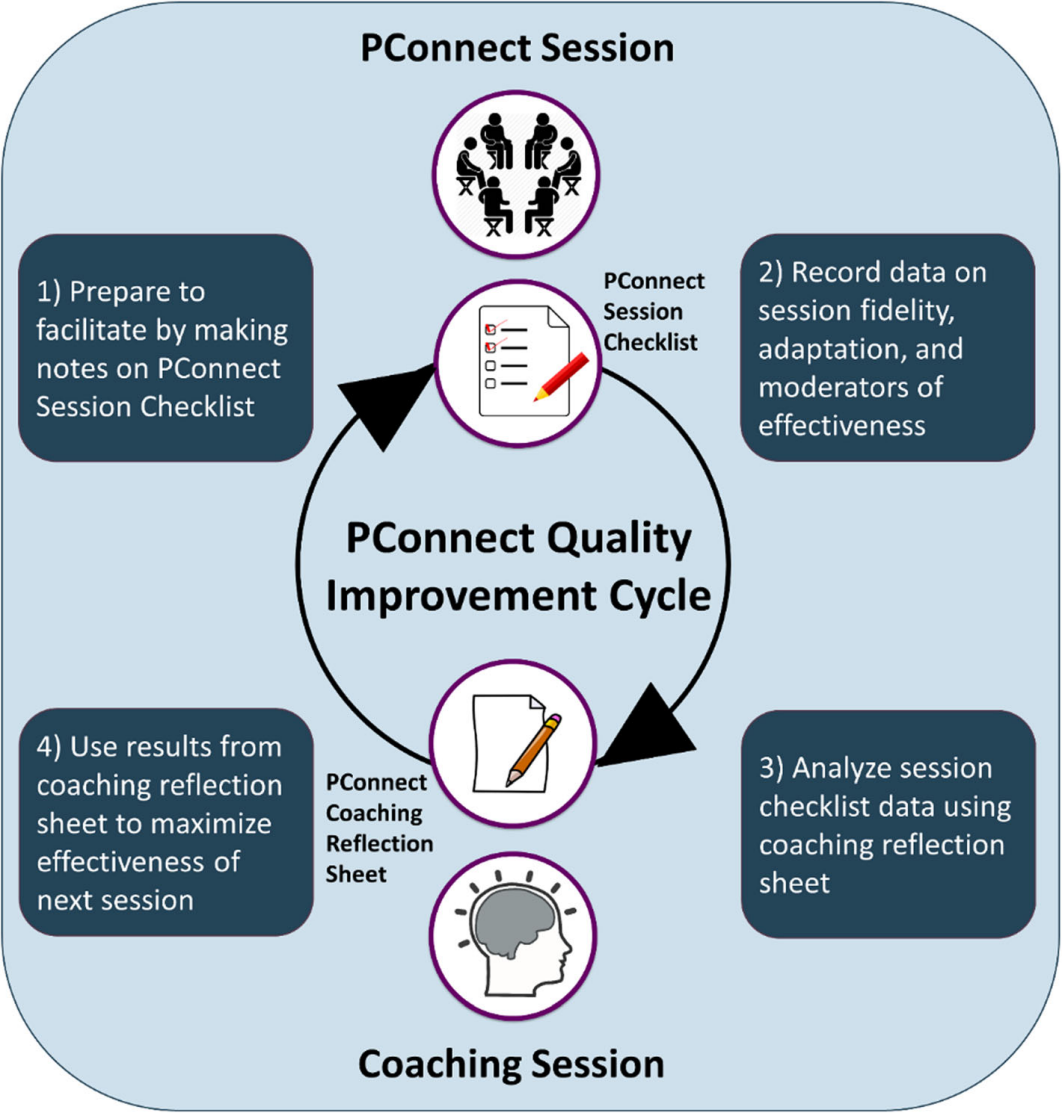
Parents Connect for Healthy Living (PConnect) quality improvement cycle. This quality improvement protocol is used during the 10-session PConnect program to maximize facilitator effectiveness. Data collected for quality improvement are also used for process evaluation

Two data collection tools, the PConnect session checklists and the coaching reflection sheet (Figs. 1 and 2), are used to collect process evaluation data as described above. They are also integral to the quality improvement cycle built into the PConnect facilitation model (Fig. 3). The quality improvement cycle begins when facilitators prepare to lead a session by reviewing the entirety of the session as detailed in a comprehensive program manual, followed by a review of the session checklist, which outlines each of the key steps to facilitate the session (Table 1, Fig. 1). The facilitators finalize their preparations by making notes on the checklist about how they plan to lead the session. The next step in the quality improvement cycle takes place during the session, when facilitators refer to the session checklist to guide them through the session. They check off each part of the session they complete and make note of any parts that they modified. Facilitators then bring their completed checklists to their weekly quality improvement coaching meeting, which is the third step of the quality improvement cycle. Coaches from the CHL research team use the coaching reflection sheet (Fig. 2) to guide facilitators through an analysis of all the data they collected on their session checklist. The final step in the quality improvement cycle is to use the results of this analysis to inform the strategies facilitators will employ during subsequent PConnect sessions to maximize their effectiveness. This quality improvement cycle parallels the Plan-Do-Study-Act cycle, a best practice in healthcare quality improvement [23].

### Data quality, storage, and analysis

In Table 1, we specify for each data collection tool when the data are collected and how they are collected (e.g., paper, electronic). Before distributing compensation for completing any of the questionnaires (tools A–E in Table 1), data completeness is checked by research team staff. If a substantial proportion of the questionnaire is blank, the participant is asked to ensure he/she did not accidentally skip anything. Research team staff are also responsible for ensuring complete data for all administrative records (tool I in Table 1) as well as PConnect observations and checklists (tools L–N in Table 1). Data from all tools are entered by research team staff and stored in a secure REDCap database. Completed paper forms are stored in a locked research office. All stored data are de-identified, using numeric identification numbers for individuals and Head Start programs. Interviews and focus groups are digitally recorded, transcribed, and translated to English if conducted in any other language. Interview and focus group data are stored in a secure computer system.

Access to all process evaluation data is restricted to the CHL research team. The data are analyzed on an ongoing basis. Process measures related to adherence to trial protocols are summarized as soon as they are collected and presented at research team meetings held weekly or biweekly. Adjustments to implementation are made accordingly to ensure adherence to the frequency, duration, and reach of major CHL elements. For example, based on review of PConnect attendance data, additional PConnect recruitment efforts were implemented for a program with low parent turnout in order to maximize adherence to the intended frequency, duration, and reach of PConnect.

Adaptations are also made to the protocol based on process evaluation. As described previously, the quality improvement cycle for PConnect includes the analysis of process evaluation data after each session, resulting in immediate updates to implementation strategies for the following session. For instance, one pair of PConnect facilitators found participants disliked having a rigid agenda for each session and therefore decided to involve participants in determining the order in which to complete activities during subsequent sessions. The other main part of the CHL protocol adapted based on process evaluation data is the content of the trainings conducted by the CHL research team. Process evaluation data for these trainings are evaluated each year and used to improve the quality of the training. For example, the content of the PConnect facilitator training has been modified each year based on session observations and to better meet needs expressed by facilitators in interviews and on survey tools.

At the conclusion of the trial, interviews and focus groups will be analyzed thematically with two or more coders analyzing the data. Qualitative findings will be triangulated with quantitative data to create a summary score for each Head Start program for adherence, adaptation, and modifiers. These summary scores will be used to provide insight into intervention outcomes as well as the barriers and facilitators to sustaining and scaling up CHL.

## Discussion

The CHL team developed a comprehensive plan for the process evaluation of CHL prior to the implementation of CHL. For each of the three major intervention components (PConnect, enhanced nutrition support, media campaign), measurements are in place to capture information on adherence, adaptation, and moderators of implementation and program effectiveness. These data will be used to summarize overall implementation of each of the intervention elements for diverse audiences. The data can be used to explain observed patterns in implementation and outcomes (e.g., variation across Head Start programs) for stakeholders in obesity prevention research. For Head Start policymakers and implementation scientists, a summary of the implementation realized and common barriers and facilitators to successful implementation can inform efforts to sustain CHL in continuing programs and initiate CHL in new programs. As such, the process evaluation protocol can shed light on intervention outcomes while also characterizing the viable validity [4] of the intervention.

The process evaluation procedure for CHL has been designed to meet the unique challenges of CBPR. The CHL team began by selecting the Pérez et al. [7] evaluation framework to guide our process evaluation design. This framework, created for adaptive interventions, matches the theoretical underpinnings of CBPR interventions because it places explicit value on adaptation rather than implicitly imposing top-down enforcement of adherence. For instance, instead of simply quantifying how many PConnect activities are implemented as designed, facilitators are given space on the PConnect session checklists to document changes made. The quality improvement coaching sessions provide opportunity to reflect on the effectiveness of those changes to optimize delivery of upcoming sessions. As such, facilitators are encouraged to deliver the PConnect program in a way that best matches their own strengths and the needs of the parents participating in their program. Even outside the context of CBPR, adaptation is inevitable and necessary when complex interventions are implemented across many sites in real-world settings [7, 8]. Furthermore, the adaptation process can increase the ability of the intervention to fit a wide range of users, facilitating adoption of the intervention [7]. Thus, our approach to measuring adherence as well as adaptation may prove useful for many types of interventions.

Another key challenge of CBPR interventions is the need to optimize both intervention effectiveness for trial participants and the scientific rigor of the trial’s design. The first strategy the CHL team used to achieve this objective was to incorporate quality improvement into intervention protocols. The quality improvement efforts aim to maximize benefit to CHL participants during the trial, as is appropriate in CBPR [24]. At the same time, the session checklists and coaching reflection sheets that are central to the PConnect quality improvement cycle provide rich process evaluation data on adherence to and adaptation of PConnect content. Since the PConnect quality improvement cycle will remain a key element of the intervention after the trial, the effects of PConnect observed during the trial are expected to be similar to the effects that would be observed in the absence of the process evaluation; the internal validity of the trial is thus uncompromised by sharing quality improvement/process evaluation data with intervention implementers. The other process evaluation strategy to maximize benefit to CHL participants was to limit data collection that would create undue burden for Head Start staff by using existing and passive data sources whenever possible.

## Conclusion

This protocol paper details the process evaluation design for a CBPR obesity intervention, answering a call for process evaluation reporting in the obesity intervention literature and offering an example of the application of an adaptive intervention evaluation framework to meet the demands of CBPR. Simultaneously, it underscores the need for (1) more widespread reporting of comprehensive process evaluation efforts, especially in the CBPR literature; (2) best practices for capturing adherence and adaptation; and (3) solutions for balancing experimental design considerations with participant benefit. In particular, it would be useful to develop best practices for working collaboratively with community partners to create process evaluation protocols that accomplish these goals. These areas of research have promise for enhancing the understanding of the factors underlying success in obesity interventions while facilitating development of obesity interventions that can be implemented across diverse settings.

## Data Availability

Not applicable.

## Abbreviations

CBPR: Community-based participatory research
CHL: Communities for Healthy Living
PConnect: Parents Connect for Healthy Living
